# Oxalate induces breast cancer

**DOI:** 10.1186/s12885-015-1747-2

**Published:** 2015-10-22

**Authors:** Andrés M. Castellaro, Alfredo Tonda, Hugo H. Cejas, Héctor Ferreyra, Beatriz L. Caputto, Oscar A. Pucci, German A. Gil

**Affiliations:** 1 Departamento de Química Biológica, Facultad de Ciencias Químicas, Universidad Nacional de Córdoba- CIQUIBIC, CONICET, Córdoba, Argentina; 2 Primera Cátedra de Ginecología, Hospital Nacional de Clínicas, Universidad Nacional de Córdoba, Córdoba, Argentina; 3 Cátedra de Patología, Hospital Nacional de Clínicas, Universidad Nacional de Córdoba, Córdoba, Argentina

**Keywords:** Microcalcifications, Oxalate, Calcium Oxalate, Breast Cancer Induction

## Abstract

**Background:**

Microcalcifications can be the early and only presenting sign of breast cancer. One shared characteristic of breast cancer is the appearance of mammographic mammary microcalcifications that can routinely be used to detect breast cancer in its initial stages, which is of key importance due to the possibility that early detection allows the application of more conservative therapies for a better patient outcome. The mechanism by which mammary microcalcifications are formed is still largely unknown but breast cancers presenting microcalcifications are more often associated with a poorer prognosis.

**Methods:**

We combined Capillary Electrochromatography, histology, and gene expression (qRT-PCR) to analyze patient-matched normal breast tissue vs. breast tumor. Potential carcinogenicity of oxalate was tested by its inoculation into mice. All data were subjected to statistical analysis.

**Results:**

To study the biological significance of oxalates within the breast tumor microenvironment, we measured oxalate concentration in both human breast tumor tissues and adjoining non-pathological breast tissues. We found that all tested breast tumor tissues contain a higher concentration of oxalates than their counterpart non-pathological breast tissue. Moreover, it was established that oxalate induces proliferation of breast cells and stimulates the expression of a pro-tumorigenic gene *c-fos*. Furthermore, oxalate generates highly malignant and undifferentiated tumors when it was injected into the mammary fatpad in female mice, but not when injected into their back, indicating that oxalate does not induce cancer formation in all types of tissues. Moreover, neither human kidney-epithelial cells nor mouse fibroblast cells proliferate when are treated with oxalate.

**Conclusions:**

We found that the chronic exposure of breast epithelial cells to oxalate promotes the transformation of breast cells from normal to tumor cells, inducing the expression of a proto-oncogen as *c-fos* and proliferation in breast cancer cells. Furthermore, oxalate has a carcinogenic effect when injected into the mammary fatpad in mice, generating highly malignant and undifferentiated tumors with the characteristics of fibrosarcomas of the breast. As oxalates seem to promote these differences, it is expected that a significant reduction in the incidence of breast cancer tumors could be reached if it were possible to control oxalate production or its carcinogenic activity.

**Electronic supplementary material:**

The online version of this article (doi:10.1186/s12885-015-1747-2) contains supplementary material, which is available to authorized users.

## Background

Cancer is one of the major public health problems of the world. Among the different types of cancer, breast cancer is one of the most frequently diagnosed one and the leading cause of cancer death in females around the world. [1, 2]. One shared characteristic of breast cancer is the appearance of mammographic mammary microcalcifications. These microcalcifications are routinely used to detect breast cancer in its early stages, which is of key importance because early detection allows the application of more conservative therapies and results in a better patient outcome. Up to 50 % of all non-palpable breast cancers are detected solely through microcalcifications observed in mammogram scans whereas up to 93 % of cases of ductal carcinoma in situ (DCIS) present microcalcifications [3]. Studies have shown that breast cancers presenting with microcalcifications are more often associated with lymph node invasion [4] and HER-2 positivity [5, 6], which results in a poorer prognosis.

Mammary microcalcifications can be classified at the molecular level in two different types well distinguished by their physical and chemical properties. -Type I calcifications that are composed of calcium oxalate (CaOx), are amber in color, partially transparent and form pyramidal structures with relatively planar surfaces. -Type II calcifications that are composed of calcium phosphate, mainly hydroxyapatite (CaP), are grey-white, opaque and form ovoid or fusiform shapes with irregular surfaces [7].

CaOx crystals have been associated both with invasive carcinomas as well with in situ lesions [8]. However, CaOx crystals are mainly related to diverse benign cystic breast lesions [9–11]. Thus, CaOx crystals in breast biopsies are often clinically significant and although it is important to detect their presence, CaOx Crystals usually are not clearly visible on routine histologic sections. Therefore, it is recommended the examination of all breast biopsies under polarized light to clearly see CaOx crystals. The mechanism by which mammary microcalcifications are formed is still largely unknown. No clear demonstration has shown if an active cellular process produces microcalcifications or if these are the result of cellular degeneration. Some results support the hypothesis that CaOx would be a secretion product whereas CaP could be formed due to an active process similar to the one involved in the physiological mineralization of bone rather than a passive, end stage process associated with cellular degeneration. Furthermore, other groups find that epithelial cells acquire mesenchymal characteristics and become capable of producing breast CaOx microcalcifications [10–12].

Oxalate has also been found as an inert metabolic end product because mammalian cells cannot metabolize it. Oxalate is an organic dicarboxylate that may be present as free oxalic acid, as soluble salts such as sodium or potassium oxalates, or as insoluble salts such as calcium oxalate crystals [13, 14]. Additionally, oxalate is produced by many kinds of cells, including liver cells, kidney, epithelial cells and apocrine cells, among others [8, 12, 15–18]. Oxalate deposits are associated with renal cysts in acquired renal cystic disease, hyperplastic thyroid glands, and benign neoplasms of the breast [19]. In breast, apocrine cells originate from the terminal duct–lobular unit and not from axillary apocrine sweat glands [15, 20]. Apical secretory snouts are usually found in cells of the apocrine metaplasia, and intra-cytoplasmic vacuoles are present. Intraluminal calcium oxalate crystals have been occasionally seen in association with apocrine metaplasia, especially in dilated ducts [20].

It is supposed that the accumulation of oxalate is toxic to living tissue since it induces some pathological circumstances, as mentioned above. Indeed, exposure of renal epithelial cells to oxalate triggers diverse events that include a plethora of cellular changes on the p38 MAPK pathway activity, induction of immediate early gene expression like *c-fos* gene and re-initiation of DNA synthesis, among others [21, 22]. Furthermore, oxalate stimulates IL-6 production in human renal proximal tubular epithelial cells [23–25]. By Affimetrix gene expression it was found 750 up-regulated and 2276 down-regulated genes in renal cells exposed to oxalate.

Despite the importance of mammary microcalcifications for the early detection of breast cancer and their potential prognostic and biological relevance, little research has been carried out to investigate its function and even more, to the best of our knowledge, no one has considered free oxalate as an important inductor of breast pathologies. Scarce research has been carried out directed to specifically investigate the impact that the presence of oxalates has on the breast tumor microenvironment. Neither the interactions between oxalate breast-epithelial cells are well understood nor have been elucidated the signal transduction pathways involved in it. Herein, we have obtained evidence supporting the hypothesis that the chronic exposure of breast epithelial cells to oxalates induces alterations in normal breast epithelial cells promoting the transformation of breast cells from normal to tumor cells.

## Methods

### Oxalate determination

Using an Ultra-turrax homogenizer, either human or murine breast tissues were processed in hydrochloric acid 2.75 M. Always a ratio of 1:5 was maintained between the milligrams of tissue and micro-liters of hydrochloric acid used, approximately 200 mg of breast tissue (tumor or not) was homogenized in 1000 μL of hydrochloric acid. Then it was centrifuged for 15 min at 15,000**x**g and the supernatant fraction (SF) was stored at −20 °C. Total Oxalate concentrations in the SF’s were quantified using capillary electrochromatography (CEC) (Beckman Coulter).

### Tissue homogenates for protein analysis

Using an Ultra-turrax homogenizer, either human or murine breast tissues were processed in RIPA buffer (NaCl 150 mM, Tris–HCl 50 mM, EDTA 0,5 mM, Tritón 1 % y SDS 0,1 %) plus complete protease inhibitor cocktail [7]. Always a ratio of 1:5 was maintained between the milligrams of tissue and micro-liters of buffer used, approximately 200 mg of breast tissue (tumor or not) was homogenized in 500 μL of buffer. This process was made on ice (4 °C) and then centrifuged for 15 min at 15,000**x**g using a cooling centrifuge (4 °C) to separate the microsomal [26] and supernatant (SF) fractions. SF was stored at −20 °C to future protein analysis by SDS page and Western Blot.

### Cell cultures and extracts

MCF-7, MDA-MB231, MCF-10A, NIH-3 T3 and HEK-293 cells (ATCC-Bethesda, MD, USA) were grown under standard culture conditions in Dulbecco’s modified Eagle medium (Gibco, BRL, Invitrogen, Carlsbad, CA, USA) supplemented with 10 % fetal bovine serum (FBS). MCF-10A cells were grown under standard culture conditions in DMEM/F12 (Gibco, BRL, Invitrogen, Carlsbad, CA, USA) supplemented with 10 % fetal bovine serum (FBS) and additionally have the following supplements: EGF 20 ng/ml, Hydrocortizone 0,5 mg/ml, Cholera Toxin 100 ng/ml, Insulin 10 μg/ml. After desired confluence, growth was continued for 48 h (MCF-7, MCF-10A, NIH-3 T3 and HEK-293 cells) or 72 h (MDA-MB231 cells) with serum-free media (−FBS) to achieve quiescence. Cells reentered growth by addition of 10 % FBS or cultures continued with serum-free media (−FBS), as indicated in the experiments. Obtaining of total homogenate (TH) from attaches cell: Cell growth medium was removed from 35 mm well and then cells were rinsed with PBS. After that, cells were lysed with 90 μL of RIPA buffer (NaCl 150 mM, Tris–HCl 50 mM, EDTA 0,5 mM, Tritón 1 % y SDS 0,1 %) plus complete protease inhibitor cocktail [7] using a scrapper and then centrifuged for 15 min at 15,000**x**g using a cooling centrifuge (4 °C) to separate the microsomal [26] and supernatant (SF) fractions. SF was stored at −20 °C to future protein analysis by SDS page and Western Blot.

### Protein quantification

Total protein concentration in the SF from TH of cells or tissues (for more details see above) was performed using Bradford standard colorimetric method (Bio-Rad protein assay).

### SDS – Polyacrylamide gel electrophoresis and western blot assays

60 μg of total protein from TH of cells or tissues (for more details see above) was subjected to SDS-gel electrophoresis according to Laemmli. The gel concentration was 12 % and the acrylamide - bisacrylamide ratio was 30 and 0.8 % p/v respectively. The separated proteins were electrotransferred to PVDF membrane (pore size 0,2 μm, Westran S, Sigma-Aldrich) at 300 mA for 1 h according to Anthony K. Tan. For immunoblotting, non-specific binding sites were blocked with PBS containing 5 % non-fat milk and Tween 20 0.05 % p/v, for 1 h at room temperature. Blocked membranes were incubated overnight at 4 °C in PBS-Tween 20 0.05 % p/v with: rabbit anti-c-Fos monoclonal antibody (Sigma-Aldrich, dilution 1/1000), rabbit anti c-Jun (Sigma-Aldrich, dilution 1/1000), mouse anti α-tubulin DM1A mAb (Sigma-Aldrich, dilution 1/5000). Washed membranes were incubated 1 h at room temperature with IRDye 680LT anti-rabbit or IRDye 800CW anti-mouse antibody (1/25,000, LI-COR Bioscience, Lincoln, NE, USA), washed and immunodetection performed using ODYSSEY Infrared Imaging System (LI-COR Bioscience).

### Real time–RT-PCR

Total RNA was extracted from breast tissue and cell lines using Trizol Reagent (Invitrogen) and an RNeasy Mini Kit (Qiagen) respectively. One microgram of total RNA was transcribed into cDNA using the SuperScript™ III First-Strand Synthesis System (Invitrogen). The qRT-PCR primers (Taqman) were purchase from Applied Biosistem. Specific transcripts were quantified by real time qRT-PCR (ABI 7500 Sequence Detection System, Applied Biosystems) using the Sequence Detection Software v1.4. The primers used to measure human and mouse *c-fos* mRNA expression were Hs04194186_s1 and Mm00487425_m1 respectively. Gene expression of human *c-fos* was normalized to GAPDH using the primer Hs99999905_m1 (MCF-7 cells) or to RPLPO using the primer Hs99999902_m1 (HEK cells). Gene expression of mouse *c-fos* was normalized to Tbp using the primer Mm00446973_m1. The relative gene expression was calculated using the 2^-ΔΔCt^ method. Each sample was analyzed in quadrupled.

### Cell proliferation assay

Cell proliferation was assessed using the CyQUANT® cell proliferation assay kit (Molecular Probes Inc., OR, USA) or counting cells with a Neubauer Chamber. In the first method, the trademarked CyQUANT® dye binds to DNA, and the fluorescence emitted by the dye is linearly proportional to the number of cells in the well. Cells were plated in 96-well black fluorescence plates, at a density of 4000 cells/well. Experiments were carried out at least three times by quadruplicated. In the counting cells method, cells were plated in 6-well plates at a density of 30,000 cells/well and also they were carried out at least three times by quadruplicated. Cells were cultured by three days at different conditions and after that, cells were trypsinized and counted by triplicate using the Neubauer Chamber.

### Ethics statement

Freshly excised human breast tumor and matched benign specimens were obtained from female patients after they were informed about all process and signed the consent accepting to participate in this study (Informed Consent). The Research Ethics Board of the Hospital Nacional de Clinicas, Universidad Nacional de Cordoba, Argentina, approved all the procedures used for this study (with the Helsinki Declaration of 1975, as revised in 1983). Samples were processed anonymously. Patient ages ranged from 38 to 82 years old.

### Animals

Female BALB/c or BALB/c nude mice (Charles River) were injected into the left inguinal mammary fatpad with 50 μL of oxalic acid 810 μM in a carrier solution containing CaCl_2_ 1.8 mM (experimental group), with carrier solution or with carrier solution plus acetic acid 810 μM. Animals received 9 injections in a period of 29 days (one injection every three or four days) of 50 μL each one. To avoid the formation of oxalate microcrystals mice were injected into the left inguinal mammary fat pad with potassium oxalate 810 μM in a carrier solution. Also the mice were injected in the back with the same solutions potassium oxalate 810 μM, acetic acid 810 μM or carrier solution. Animals received 7 injections in a period of 18 days (one injection every two or three days) of 50 μL each one. When tumors were over 1000 mm^3^, mice were euthanized for tissue collection or when the animals were in no healthy condition. All animal breast tissues were stained for H&E. Two independent pathologists made histopathological analyses. All mice were grown under standard conditions. The Ethics Committee of the Department of Chemical Biology, UNC, Argentina, approved all the procedures used for this study.

### Macrodissection

To perform macrodissection, 3–5 serial 10-μm sections of tumor were adhered to uncharged slides using nuclease-free water. One additional 5-μm adjacent section was stained for H&E. An expert breast histopathologist outlined the tumor hotspot region. Each slide from the block was then overlaid on the H&E-stained slide and oriented according to the features of the section. The area surrounding the tumor-dense target region was scraped away using a sterile razor blade; the remaining tumor region was scraped into a 1.7-ml tube using a fresh blade. This process was repeated for all of the sections for each macro-dissected sample.

### Immunohistochemistry

Breast Tumor Tissue specimens were de-waxed and re-hydrated as described [27] and incubated overnight at 4 °C with rabbit anti-c-Fos monoclonal primary antibody (Sigma-Aldrich, dilution 1/300) diluted in blocking buffer. Sections were rinsed 3 times with PBS 10 mM plus 0.1 % Tween 20 (PBS-tween) and then incubated with anti-rabbit Alexa 488 secondary antibody (dilution 1/500, Molecular Probes, Eugene, OR, USA) for 2 h at RT. Sections were rinsed three times with PBS-tween and nuclei were stained by incubation with 4′,6-diamidino-2-phenylindole (DAPI) 20 min and rinsed again with Milli-Q water. Slides mounted with FluorSave (Calbiochem, San Diego, CA, USA) were visualized under an Olympus FV1000 or Pascal 5 laser scanning confocal microscope using Olympus (Centre Valley, PA, USA) or Carl Zeiss (St Louis, MO, USA) software for image analysis.

### Statistical analyses

**Fig. 1:**
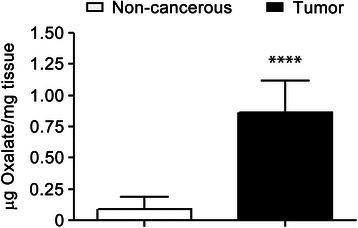
Human breast tumor tissues have higher concentration of total Oxalate than non-cancerous breast tissues. 13 samples of human breast tumor tissues (Tumor samples) and 12 samples of tumor-adjacent non-cancerous breast tissues (Control samples) were homogenized in 2.75 M hydrochloride acid. The supernatant fractions were analyzed by Capillary Electrochromatography to establish the total concentration of oxalate present in each sample. Results of oxalate concentrations are expressed as μg oxalate/mg of tissue (*n* = 13 and *n* = 12 were analyzed for tumor and non-tumor samples, respectively); statistical significance was calculated using Student’s two tailed *t* -test. ****P value < 0.0001

**Fig. 2:**
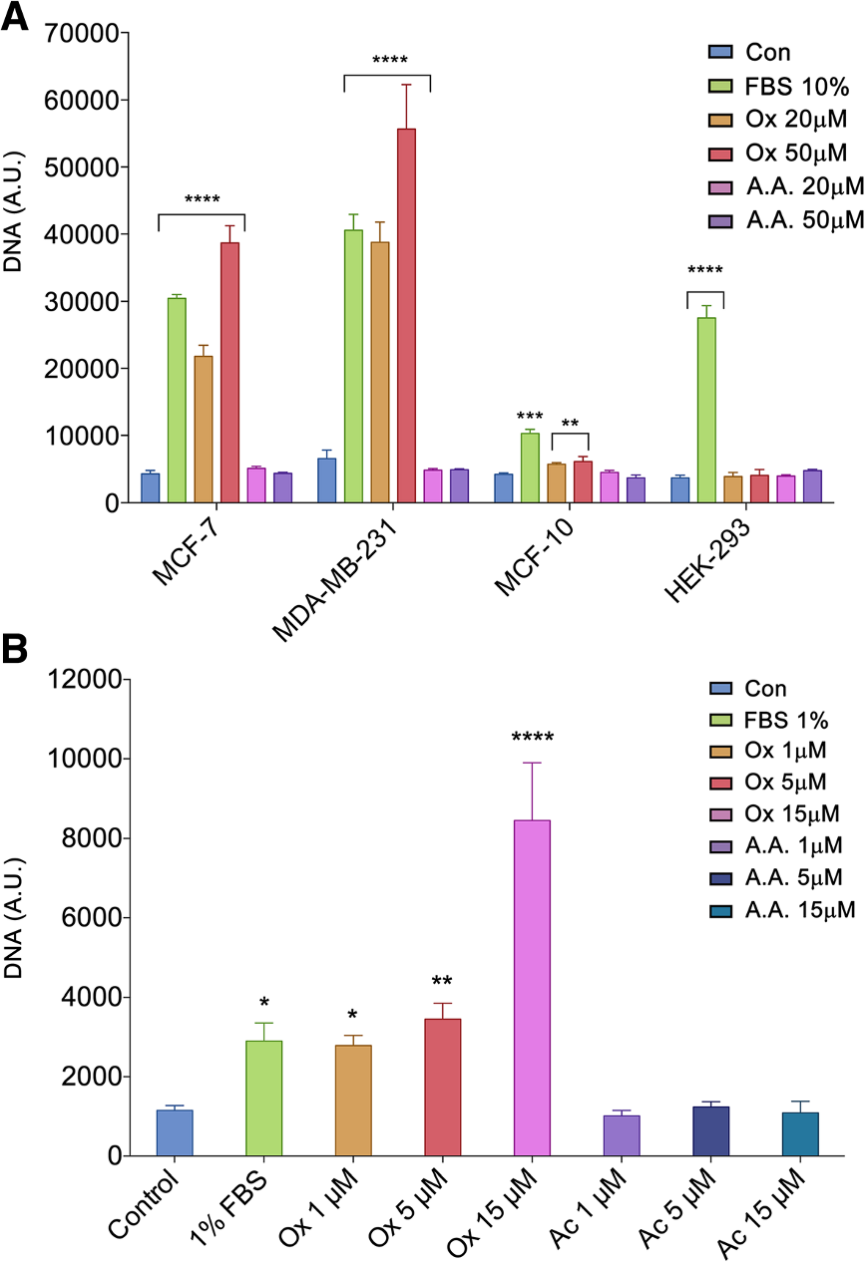
Oxalate induces breast cancer cell proliferation. Proliferation was performed using a colorimetric assay and following the manufacturing instructions (CyQUANT, Life Technologies) in cells (**a**) after 3 days of treatment of MCF-7, MDA-MB231, HEK-293 or 7 days of treatment of MCF-10A cells (**a**) as indicated with oxalate or with acetic acid, or (**b**) after 3 weeks of treatment in of MCF-7 cells. Cells were cultured in DMEM medium plus an additional specific reagent or not, according to each condition, as indicated. Con: Control, no additional reagent was added; FBS: fetal bovine serum, Ox: oxalic acid or A.A.: acetic acid were added to the culture medium. Results are expressed as DNA content (arbitrary units) found after seeding 4 x10^3^ cells/well.. Bars represent the standard error of the mean of four independent experiments performed in triplicate. A.U.: arbitrary units. Statistical significance determined by Two-way ANOVA with Holm-Sidak’s multiple comparison test (α = 0.05) were performed in experiments graphed in Fig. 2a. One- way ANOVA with Holm-Sidak’s test was performed in Fig. 2b. ****:*P* value <0.001; **P value <0.01; *P value < 0.05

Each statistical analysis applied to the results has been clarified in the corresponding legends to the figure. Survival curves of Fig. 5 were statistically analyzed by LogRank (Mantel-Cox) test whereas statistical significance analysis by Two-way ANOVA with Holm-Sidak’s multiple comparation test (α = 0.05) was performed in experiments graphed in Fig. 2a and Additional file 1: Figure S1. One- way ANOVA with Holm-Sidak’s test was used in Figs. 2b, 4c and d [28]. Figures 1 and 6 were statistically analyzed by Student’s two-tailed *t* -test. All graphs were performed using GraphPad Prism version 6.0e for Mac OS X (GraphPad Software, La Jolla California USA).

## Results

### Oxalate levels are increased in human breast tumor tissues

In order to study the biological implications of Oxalates within breast tumors, first oxalate concentration was determined using Capillary Electrochromatography (CEC) in both human breast tumor tissues and tumor-adjacent non-cancerous breast tissues. Eleven breast tumor samples (Tumor samples) and the same numbers of non-cancerous breast tissues (Control samples) were analyzed. Most of the paired-samples were obtained from a same patient and each patient was randomly selected. Surprisingly, we found that all breast tumor tissues examined have a higher concentration of total oxalate than their counterpart non-cancerous breast tissue. The average concentration of total oxalate present in the tumor samples was almost 10 times higher than that of the control samples. A significant difference between tumors vs. control was found after analyzing results for statistical significance using Student’s two tailed *t* test (Fig. 1).

### Oxalate induces proliferation of breast cells

Due to the high concentration of oxalate found in breast tumor tissues relative to breast non-tumor tissues, we hypothesized that oxalate could produce a particular effect at the cellular level that would favor the genesis and growth of breast tumors. Therefore, breast cancer cell lines were treated in culture with different concentrations of oxalate and then proliferation was measured. Cell proliferation assays were performed using two different techniques, that is, measuring total DNA (Fig. 2a) and counting cells (Additional file 1: Figure S1). The experiments were performed three times in quadruplicate.

These proliferation experiments showed that oxalate at concentrations of 20 and 50 μM induces significantly higher rates of proliferation of MCF-7, MDA-MB231 cell lines after three days of treatment as determined by Two-way ANOVA with Holm-Sidak’s multiple comparison test (α = 0.05) (Fig. 2a). Furthermore, MCF-7 cells treated for three weeks with lower concentrations of oxalate (1 μM, 5 μM and 15 μM) also showed a significant increase in their proliferation rates as determined by One- way ANOVA with Holm-Sidak’s test (Fig. 2b). It is important to note that in all cases the effect of oxalate was evaluated using the same culture medium as used for the corresponding control. To exclude any possible involvement of the pH in this phenomenon, for each case acetic acid was used as an additional control, at the same concentration as the oxalate. As expected, none of the acetic acid concentrations had any significant effect on cell proliferation as shown in Fig. 2a and b. Similar controls were performed with fumaric acid and essentially the same results were obtained (not shown). Interestingly, oxalate has no effect on other cell types, such as HEK-293 cells (Fig. 2a) or NIH-3 T3 cells (Additional file 1: Figure S1), indicating that this phenomenon is restricted mainly to breast cells. However, normal breast cell lines such as MCF-10A cells were treated for three days with oxalate (at 20 μM or 50 μM) but proliferation induction was not clearly observed (results not shown). Consequently, MCF-10A cells were treated with oxalate for a longer period of time (seven days) with the same concentrations that above (Fig. 2a). Under these latter experimental conditions of prolonged treatment with oxalate, a slight induction of proliferation was observed in normal breast cells line but of a smaller magnitude than the induction promoted by treatment of these cells with FBS plus EGF. Furthermore, in no case was this induction of a similar magnitude to that obtained after treatment of the breast cancer cells lines with FBS or with oxalate (Fig. 2a). This was the first indication that oxalate has a proliferative effect on breast cells.

### Oxalate induces overexpression of c-Fos in MCF-7 cells

**Fig. 3:**
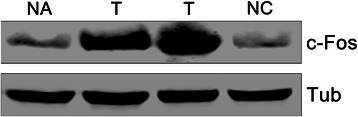
c-Fos is over-expressed in human breast tumor tissues. Western Blot of Breast tumor tissue (T) and paired tumor-adjacent non-cancerous tissue (NA) from a patient, together with a non-paired non-cancerous breast tissue (NC) (as an additional control) were fractionated in 12 % SDS-PAGE gel and immunoblotted using anti-c-Fos and anti α-Tubulin antibodies. α-Tubulin was used as loading control. The blot shown is representative of five independent experiments performed that gave similar results

**Fig. 4:**
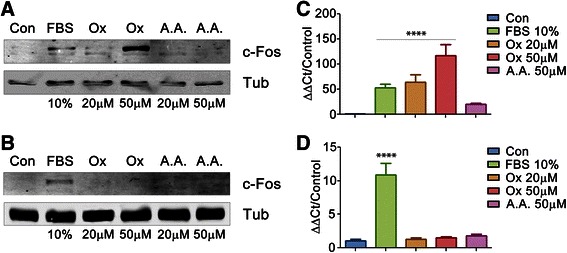
Oxalate induces expression of c-Fos in MCF-7 cells but not in HEK-293 cells. Cells were plated in six wells and grown to 80 % of confluence. Then cells were starved to achieve quiescence (see M & M). After that, each experimental condition was achieved by the stimulation with the specified reagent during 1.5 h. MCF-7 (**a**) or HEK-293 (**b**) cells were lysed, then the supernatant fractions were separated in 12 % SDS-PAGE gel and immunoblotted using anti-c-Fos antibody. α-Tubulin was used as loading control. *c-fos* expression was measured in MCF-7 (**c**) or HEK-293 (**d**) cells by qRT-PCR and normalized against housekeeping genes (GAPDH for MCF-7 cells and RPLPO for HEK-293 cells) using the Sequence Detection Software v1.4. Shown are the mean values of 3 independent determinations performed in quadruplicate. Con: Control, No reagent addition FBS: fetal bovine serum. Ox: oxalic acid. A.A.: acetic acid. Statistical significance determined by One- way ANOVA with Holm-Sidak’s test was performed in experiments shown in Fig. 4c and d, **** *P* value < 0.0001

*fos* and *jun* oncogenes are members of the family of Immediate Early Genes (IEGs) that are rapidly and transiently expressed in different cell types in response to a myriad of stimuli, such as growth factors, neurotransmitters, etc. [19, 27, 29–32]. Although c-Fos was described as an AP-1 transcription factor more than 25 years ago, the complex consequences of its induction on cell’s physiology have still not been fully elucidated. It has been proposed that, upon mitogenic stimuli, c-Fos triggers and controls cell growth, differentiation and apoptosis by regulating key genes. Furthermore, c-Fos was also described as a cytoplasmic activator of the biosynthesis of lipids both in normal and pathological cellular processes that demand high rates of membrane biogenesis [33]. c-Fos and Its family members are probably the most frequently expressed IEGs in different forms of human cancer: its overexpression has been reported in proliferative disorders such as breast, lung, colon, brain and thyroid cancers [27, 30, 34]. We analyzed c-Fos expression by Western Blot in both human breast tumor tissues and non-cancerous breast tissues adjacent to tumors. Both types of tissues were taken in pairs from patients. As expected, we also observed high rates of c-Fos expression in breast tumor tissues, whereas non-cancerous tissues showed little or non-detectable levels of c-Fos expression confirming the results of Motrich et al. [34] (Fig. 3). Consequently, we evaluated the possible effect of oxalate on breast cancer cell lines in terms on inducing c-Fos expression. This evaluation was done by exposing MCF-7 cells to different concentrations of oxalate for 1.5 h and then c-Fos expression was measured by Western Blot (Fig. 4a). c-Fos over-expression is induced by oxalate in a concentration range between 20 and 50 μM in MCF-7 cells. This result reveals for the first time that oxalate can activate the pathway of an IEG such as *c-fos*, exhibiting a genomic effect, in breast cancer cells. Conversely, HEK-293 (Fig. 4b) and NIH-3 T3 (Additional file 2: Figure S2) cells were treated with oxalate in parallel to MCF-7 cells, but neither non-breast cell lines over-expressed c-Fos showing that this effect is limited to breast cells. This result is in accordance with the previous one, in which it is showed that the effect of oxalate on proliferation is observed mainly in breast cells. We also tested the induction of other IEG as c-Jun by oxalate in MCF-7 cells in the same condition that above. c-Jun expression was not induced by oxalate as its shown in the Western Blot in Additional file 3: Figure S3.

To further confirm that oxalate can activate the pathway of *c-fos* in MCF-7 cells but not in HEK-293 cells, we measured the transcription level of this gene by q-RT-PCR. For this, both cell lines were treated as described previously for 1.5 h with oxalate and then *c-fos* mRNA measured. As expected, over-expression of *c-fos* mRNA was found in MCF-7 cells treated with oxalate (Fig. 4c) but not in HEK-293 cells (Fig. 4d).

### Oxalate induces breast tumors in mice

**Fig. 5:**
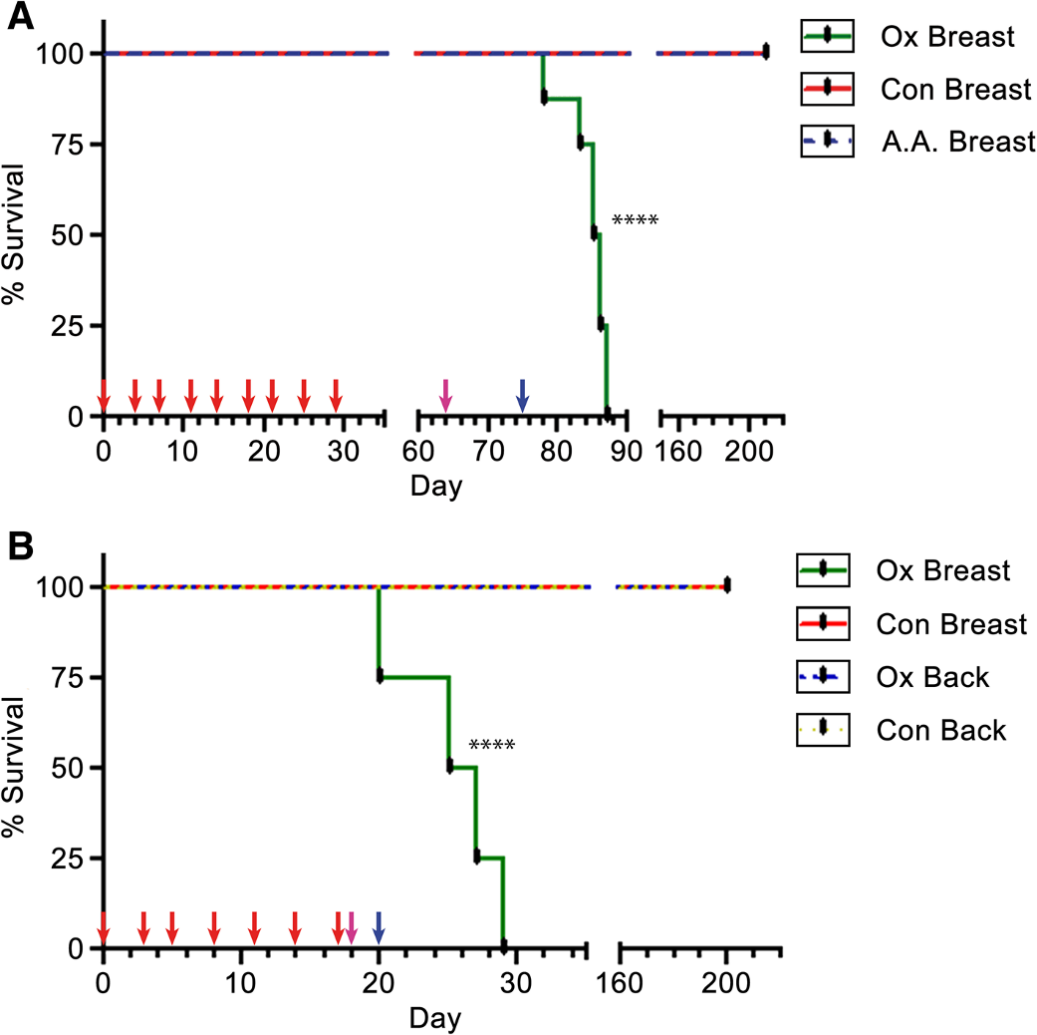
Oxalate induces tumor formation and short-term survival in mice. **a** Mice were injected every 3 or 4 days into mammary fat pad to reach 9 doses (one dose every 3.2 days on average). The experimental group (Ox Breast) received 50 μL of a solution with microcrystals of calcium oxalate (oxalic acid 810 μM in a carrier solution containing CaCl_2_ 1.8 mM). The two control groups received 50 μL of either carrier solution containing CaCl_2_ 1.8 mM (Con Breast) or a solution of carrier solution plus acetic acid 810 μM (A.A. Breast). Each group consisted of 8 mice. **b** Mice were injected every 2 or 3 days to reach 7 doses (one dose every 2.6 days on average). Experimental groups received injections with 50 μL of saline solution containing potassium oxalate 810 μM either at the mammary fat pad (Ox Breast) or in the Back (Ox Back). Control groups were injected at the same places as the experimental ones with saline solution only into mammary fat pad (Con Breast) or into the back (Con Back). Each group consisted of 5 mice. Red arrows: times of injection. Pink arrows: time of the first tumor appearance in Ox Breast group. Blue arrows: time at which all mice of Ox breast group have at least one tumor. Statistical significance between survival curves was analyzed by LogRank (Mantel-Cox) test. ****P value < 0.0001

**Fig. 6:**
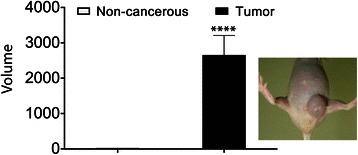
Oxalate-treated mice generated tumors. Mice were injected every 2 or 3 days with 50 μL of saline solution containing oxalate 810 μM at into mammary fat pad. Control groups were injected at the same places than the experimental ones with saline solution. Tumor volume (v) of the mice treated were measured and calculated in accordance with the method of Attia and Weiss [35]. To the right it has been included a photograph of a typical tumor induced by oxalate in mice. Each experimental group consisted of 9 animals per group. Results were analyzed for statistical significance using Student’s two tailed *t* -test. ****P value < 0.0001

In order to well define if oxalate triggers breast cancer in vivo, we treated mice with oxalate, mimicking in vitro proliferation experimental conditions described before. Consequently, eigth mice were injected into the left inguinal mammary fat pad (mammary fat pad) with 50 μL of a solution containing microcrystals of calcium oxalate at a concentration of 810 μM (experimental group). To obtain the microcrystals of calcium oxalate, 1.8 mM CaCl_2_ was added to the solution of oxalic acid previous to being injected into the mice. Two additional groups (six mice each) were injected either with 50 μL of carrier solution plus acetic acid 810 μM or 50 μL of carrier solution alone, also into the mammary fat pad region (control groups). The mice that received injections containing acetic acid were an extra control to exclude any possible effect of pH or injury due to the frequency of injections used. Altogether, the mice received nine doses (one injection every 3 or 4 days) in a period of 29 days (1 dose every 3.2 days on average). After the last dose was applied, mice were examined and no tumor formation was observed. However on day 64 (35 days after the last dose was injected) a palpable tumor appeared at the mammary fat pad region in a mouse of the experimental group. On day 73, most mice of this experimental group had at least one tumor (6 of 8 mice), at the mammary fat pad region. Finally, on day 75, all mice injected with oxalate had developed tumors at the mammary fat pad area and some of them presented palpable metastatic tumors in the chest region. The survival curves of Fig. 5a clearly show the statistically significant differences between survival curves as analyzed by LogRank (Mantel-Cox) test. Tumor volume (v) of each mice was measured and calculated in accordance with the formula of Attia and Weiss [35], v = 0.4 x (a x b^2^), in which (a) is the largest and (b) is the smallest diameter of each tumor. All mice injected at the mammary fat pad area with oxalate had developed tumors but not those of the two other groups (Fig. 6). The volume of tumors originated after oxalate treatment vs. the volume of non-pathological samples of the controls (volume = mm^3^) was highly significant as analyzed using Student’s two tailed *t* -test (Fig. 6). Subsequently, oxalate-treated mice were sacrificed at different times once they were not healthy according to ethical conditions (see Materials and Methods) and a histological examination of the organs was performed. All organs such as kidneys, lungs, liver, spleen and bowels were found pathologically normal. Control mice were subjected to a 6-month follow-up, time at which they were examined in vivo and then sacrificed to perform a histological examination of all organs. No tumor formation was found in any of the control mice after performing both types of examinations (Figs. 5a and 6).

In the above-mentioned experiment in vivo*,* the experimental group received injections containing oxalate 810 μM. However, under these experimental conditions, most of the oxalate is in the microcrystal state as calcium oxalate and the concentration of free oxalate is only of 2.5 μM. Although this concentration is low, we previously showed that even 1 μM of oxalate is capable of inducing breast cells to proliferate in vitro after 3 weeks of treatment (Fig. 2b). Consequently, a new series of experiments were conducted in which six mice of the experimental groups were injected with potassium oxalate 810 μM in saline solution to avoid the formation of oxalate microcrystals in the solution injected. Additionally, saline solution was used as control and both, potassium oxalate solution and carrier solution were injected either in the fat pad or in the back of the animal to evaluate tissue-specificity of oxalate to generate tumors (*n* = 6, each animal group). Mice received seven doses, one injection every 2 or 3 days in a period of 18 days (a dose every 2.6 days on average). Surprisingly, the group of mice injected with oxalate solution at the mammary fat pad area was the only group that generated tumors. On day 18, a mouse of this group (1 of 5 mice) had already generated one tumor at the mammary fat pad and on day 20, all mice had at least one tumor and three of them had generated several tumors in the chest region. On the other hand, none of the mice of the others three groups developed tumors, that is, neither the mice injected with potassium oxalate or carrier solution into the back nor the mice injected with carrier solution into the mammary fat pad. These groups were kept under observation up to six months after the last injection, time at which tumor formation was not observed. After that, all mice were euthanized and the absence of tumor formation in all organs was confirmed in the control groups by histological analysis (Fig. 5b). Statistical analysis of the survival curves of the animals performed using the LogRank (Mantel-Cox) test showed that the survival times of the animals treated with oxalate in the mammary area was significantly shorter than that of the animals that received oxalate in the animal’s back (no tumor) or those receiving carrier solution alone (Fig. 5a and b).

This is the first demonstration the oxalate produces breast cancer tumors in vivo and also promotes short-term survival. Moreover oxalate tissue specificity was observed indicating that there is something in the breast cells necessary for oxalate to induce tumor formation since no tumors were formed when oxalate was injected in the mice back, nor was fibroblast cell proliferation observed when oxalate was added to the culture medium (Fig. 2a and Additional file 1: Figure S1).

### Analysis of mice’s breast tumors generated by oxalate

**Fig. 7:**
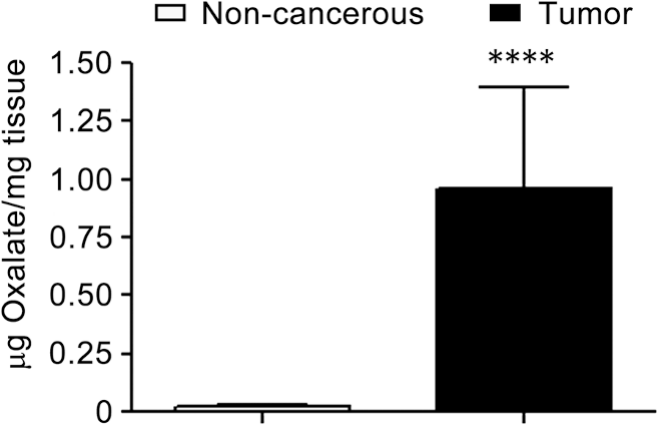
Mice breast tumor tissues contain higher concentrations of total Oxalate than non-cancerous breast tissues. Seven samples of mice breast tumor tissues (Tumor samples) and seven samples of non-cancerous breast tissues (Control samples) were homogenized in 2.75 M HCl. The supernatant fractions were analyzed by Capillary Electrochromatography to establish the total concentration of Oxalate present in each sample. Results expressed as μg/mg tissue were analyzed for statistical significance by Student’s two tailed *t* -test. All graph were performed using GraphPad Prism version 6.0e for Mac OS X (GraphPad Software, La Jolla California USA) ****P value <0.001

The concentration of oxalate was quantified by CEC in seven samples of mice’s breast tumor tissues, which were developed after oxalate treatments in vivo, and in seven samples of non-cancerous breast tissues of control mice. As expected, breast tumor tissues had higher concentrations of oxalate than non-cancerous breast tissues (Fig. 7). Additionally, sections of the mammary fat pad tumors were stained using H&E. Two independent Pathologists reported that the neoplastic growth that was found corresponds to a highly malignant lineage, undifferentiated, with characteristics of Fibrosarcoma of the breast. In these tumors, cells generally adopt a pattern arranged in fascicles of spindle cells, integrated sharp ends and large ovoid nuclei currents. In other areas of the tumor, cells are polyhedral with acidophilic cytoplasm, a highly pleomorphic angular nuclei with macronucleoli and numerous nucleoli. Upon examination of samples at under a microscope using a 40X magnifier, high mitotic activity was found; over five figures per 40X field, and atypical mitosis were also observed. Interestingly, in the breast ducts, multinuclear and abnormal epithelial breast cells were found. Immunohistochemistry of the tumors showed a marked positivity for Vimentin and low positivity to S100 protein, cytokeratin E1 to E3 and MSA (mammary serum antigen).

**Fig. 8:**
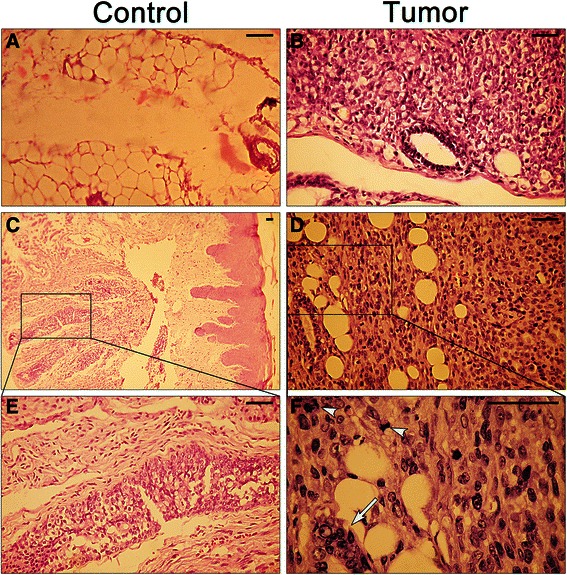
Oxalate-treated mice generated undifferentiated and highly aggressive tumors. H&E staining of two representative sections of both non-cancerous breast tissue (**a** and **b**) and breast mice tumor tissues (**d** and **e**), generated after treatment with oxalate, are shown. Panels **c** and **f** are enlargements of the areas delimited in panels **b** and **e** respectively. Panel **f** mitotic figures (*arrowheads*) and abnormal epithelial breast duct cells (*arrow*) are marked. A total of 13 tumors and of 13 non-tumor tissues were examined. In all cases, animals treated with oxalate generated malignant, undifferentiated tumors with the characteristics of fibrosarcoma of the breast. Scale bar 50 μm

In the breast tissues of all control groups (Double-Blind histological sections) of both experiments in vivo (mice treated with carrying solution or acetic acid at the mammary fat pad and those mice treated with oxalate or carrying solution at the back region) no tumor cells were found by H&E (Fig. 8).

**Fig. 9:**
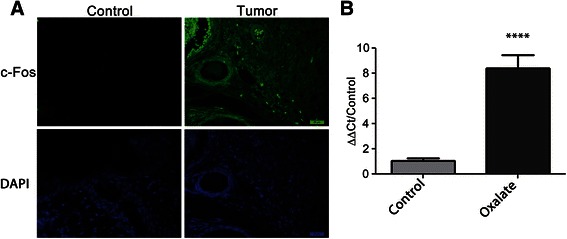
Mice breast tumor tissues express higher amounts of c-Fos than non-cancerous breast tissues. **a** Sections of both breast tumor tissues (*n* = 6) and non-cancerous breast tissues (*n* = 6) were adhered to uncharged slides. c-Fos expression was seen by immunohistochemical staining (*green*) and nuclei were visualized with DAPI (*blue*). Shown is a representative figure of all slides analyzed. A minimum if 3 sections were examined per tissue sample. **b** *c-fos* expression was measured in breast tumor tissues (*n* = 5) and non-cancerous breast tissues (*n* = 5) by qRT-PCR and normalized against the housekeeping gene Tbp using the Sequence Detection Software v1.4. A difference in c-fos expression was statistically analyzed by Student’s two tailed *t* -test. ****P value <0.001

On the other hand, sample sections from mice breast tumors (generated after treatment with oxalate) and non-cancerous breast tissues of control mice were examined by immunohistochemistry. In breast sections was seen that tumor tissues express significantly higher amounts of c-Fos than non-cancerous breast tissues (Fig. 9a). To further confirm this, these samples were analyzed by q-RT-PCR for c-fos expression. The same result was observed, tumor samples express higher levels of c-Fos m-RNA than breast control samples (Fig. 9b).

## Discussion

In the present study, the capacity of free oxalate to induce proliferation of MCF-7 and MDA-MB231 breast cancer cell lines has been demonstrated for the first time. Furthermore, it has also been demonstrated that oxalate slightly induces proliferation in a normal breast cell line such as MCF10A. In the same line, all the Balb/c nude mice that receive injections containing oxalate at the mammary fat pad region generate breast tumors showing a marked carcinogenic effect of oxalate. Different periods of time were required for each treatment to promote breast cancer and such times were directly proportional to the effective concentration of free oxalate in the solution injected. A higher concentration of free oxalate produces breast tumors more quickly. This correlation between the concentration of free oxalate and the velocity to generate breast tumors strongly supports the role of oxalate as a carcinogen chemical agent for breast tissue. Furthermore, clear differences were observed between the survival time and also between the size of tumors originated in the groups of animals that were treated with oxalate in the mammary area versus control animals treated with oxalate in the back or receiving carrier solution alone. The neoplastic growth that was found corresponds to a highly malignant, undifferentiated lineage with characteristics of a fibrosarcoma of the breast. Interestingly, in breast ducts it was observed multinuclear and abnormal epithelial breast cells. Immunohistochemistry of the tumors showed a marked positivity for Vimentin and low positivity to S100 protein, cytokeratin E1 to E3 and MSA that is compatible with the tumor described. Our working hypothesis is that oxalate is inducing an epithelial breast cell transformation such as the epithelial- mesenchymal transition. The capacity of oxalate to produce breast cancer is also supported by in vitro experiments in which different concentrations of oxalate, in a range between 20 to 50 μM, induce proliferation of human breast cell lines. Furthermore, concentrations of oxalate lower than 20 μM cause a similar effect on proliferation after a longer exposure time. The fact that oxalate can induce human breast cancer cells to proliferate in vitro is not minor. It is interesting to try to extrapolate the carcinogenic effect of oxalate in mice to a possible effect in human. It is important to highlight that we have obtained similar results to those of MP Morgan et al. 2012 [11], in which microcrystals of calcium oxalate did not induce proliferation of the breast cancer cell lines MCF-7 and MDA-MB23. We have seen that only oxalate as the free ion, has a carcinogenic potential, but not calcium oxalate crystals. Moreover, oxalate induced tumor formation only when it was injected into the breast tissue of mice but not when injected into the back of the animals, indicating that it does not induce cancer formation in any type of tissue. Actually, we do not know if oxalate can induce tumor formation in tissues other than breast tissue. In this respect, the ability of oxalate to induce cells to proliferate was only seen with breast cell lines. That is, neither human kidney epithelial cells (HEK293) nor mouse fibroblast cells (NIH/3 T3) proliferate when treated with oxalate. These are strong signals that lead us to hypothesize that the carcinogenic potential of oxalate is specific for breast tissue, although more experiments with a broader range of tissues are needed to confirm this. It is evident that the immune system plays a very important role in tumor formation. Balb/c nude mice were more sensitive to the treatment with oxalate than mice with a wild type immune system (BALB/c mice). When BALB/c mice were treated with oxalate using the same experimental conditions, no tumor development was observed at the same period of time. Furthermore, we maintained the animals under observation for more than six-months and no tumor formation was observed either. We only observed a little swelling of the mammary fat pad area in some animals a week after the last injection with oxalate solution but this swelling disappeared within 24 to 48 h (data not shown). We believe that oxalate could have initiated tumor formation although they never generated a palpable, fully developed tumor probably because the immune system response was sufficient to destroy these cells.

## Conclusions

In the present study the capacity of free oxalate to induce breast cell lines proliferation in vitro has been demonstrated for the first time. MCF-7, MDA-MB231 and MCF-10A cells treated with oxalate at concentrations of 20 and 50 μM clearly showed higher rates of proliferation than their respective controls. Furthermore, the carcinogenic capacity of oxalate was observed only when injected into the breast area of mice. In in vivo experiments, oxalate induced tumor formation when it was injected periodically into the breast tissue of Balb/c nude mice with a 100 % of penetrance. It is important to note that treatment of mice with potassium oxalate induced tumor formation more rapidly than the treatment with micro crystals of calcium oxalate although the final concentration of oxalate was the same in both cases. Probably these differences are due to the fact that calcium oxalate is poorly soluble and the concentration of free oxalate, as ion, in the equilibrium is very low. On the other hand, potassium oxalate is highly soluble and all oxalate exists in its ionic form in solution at the concentrations used. Therefore, we conclude that free oxalate, as a ion, is the chemical specie that has the carcinogenic effect on breast tissue. The tumors generated were highly malignant, undifferentiated and with the characteristics of fibrosarcomas of the breast. Moreover, we have demonstrated the ability of free oxalate to induce the expression of an IEG as c-Fos in MCF-7 breast cancer cells in vitro. Previously, it had been reported the ability of oxalates to produce changes in the expression level of many genes in a human kidney cell line [21] but this is the first time that an effect of oxalates was demonstrated in a human breast cell line. The mechanism by which oxalate exerts its action on breast cells is still largely unknown and further research is needed to elucidate it. However, it is expected that a significant reduction in the incidence of breast cancer tumors could be reached if it were possible to control the oxalate production or its carcinogenic activity.

## Additional files

Additional file 1: Figure S1. Oxalate induces proliferation of breast cancer cells but not of HEK-293 and NIH-3 T3 cells. Proliferation was measured in MCF-7, MDA-MB231, HEK-293 and NIH-3 T3 cells after 3 days of treatment by counting cells in a Neubauer Chamber. Cells were cultured in DMEM medium plus an additional specific reagent or not, according to each condition. Con: Control, none additional reagent. FBS: fetal bovine serum. Ox: oxalic acid. A.A.: acetic acid. Bars represent the standard error of the mean of three independent experiments performed in triplicate. Statistical significance determined by Two-way ANOVA with Holm-Sidak’s multiple comparison test (α = 0.05) was performed in experiments graphed in Additional file 1: Figure S1. **** P < 0.0001. (TIFF 2135 kb)
Additional file 2: Figure S2. Oxalate does not induce c-Fos expression in NIH-3 T3 cells. Cells were plated in six wells and growing up until 80 % of confluence. Then cells were starved to achieve quiescence (see Material & Methods). After that, cells were stimulated during 1.5 h with a specific reagent according to each condition. NIH-3 T3 cells were lysed and the supernatant fractions were separated in 12 % SDS-PAGE gel and immunoblotted using anti-c-Fos antibody. α-Tubulin was used as loading control. Con: Control, none additional reagent. FBS: fetal bovine serum. Ox: oxalic acid. A.A.: acetic acid. (TIFF 130 kb)
Additional file 3: Figure S3. Oxalate does not induce c-Jun expression in MCF-7 cells. Cells were plated in six wells and growing up until 80 % of confluence. Then cells were starved to achieve quiescence (see M & M). After that, cells were stimulated during 1.5 h with a specific reagent according to each condition. MCF-7 cells were lysed and the supernatant fractions were separated in 12 % SDS-PAGE gel and immunoblotted using anti-c-Jun antibody. α-Tubulin was used as loading control. Con: Control, none additional reagent. FBS: fetal bovine serum. Ox: oxalic acid. A.A.: acetic acid. (TIFF 132 kb)

## Abbreviations

CaCl_2_: Calcium chloride
CaOx: Calcium oxalate
CaP: Hydroxyapatite
qRT-PCR: Quantitative- real time -PCR
CEC: Capillary electrochromatography
DCIS: Ductal carcinoma in situ
EGF: Epidermal Growth Factor
FBS: Fetal bovine serum
HCl: Hydrochloric Acid
H&E: Hematoxylin and eosin stain
IEG: Immediate early gene
mA: Milliampere
mAb: Monoclonal antibody
MAPK: Mitogen-activated protein kinase
MF: Microsomal fraction
mM: Millimolar
μM: Micromolar
SDS: Sodium dodecyl sulfate
SF: Supernatant fraction
TH: Total homogenate
