# Supplementary figures and images for: Oxalate induces breast cancer
Source: BMC Cancer. 2015 Oct 22;15:761. doi: 10.1186/s12885-015-1747-2 (PMC4618885; doi:10.1186/s12885-015-1747-2)

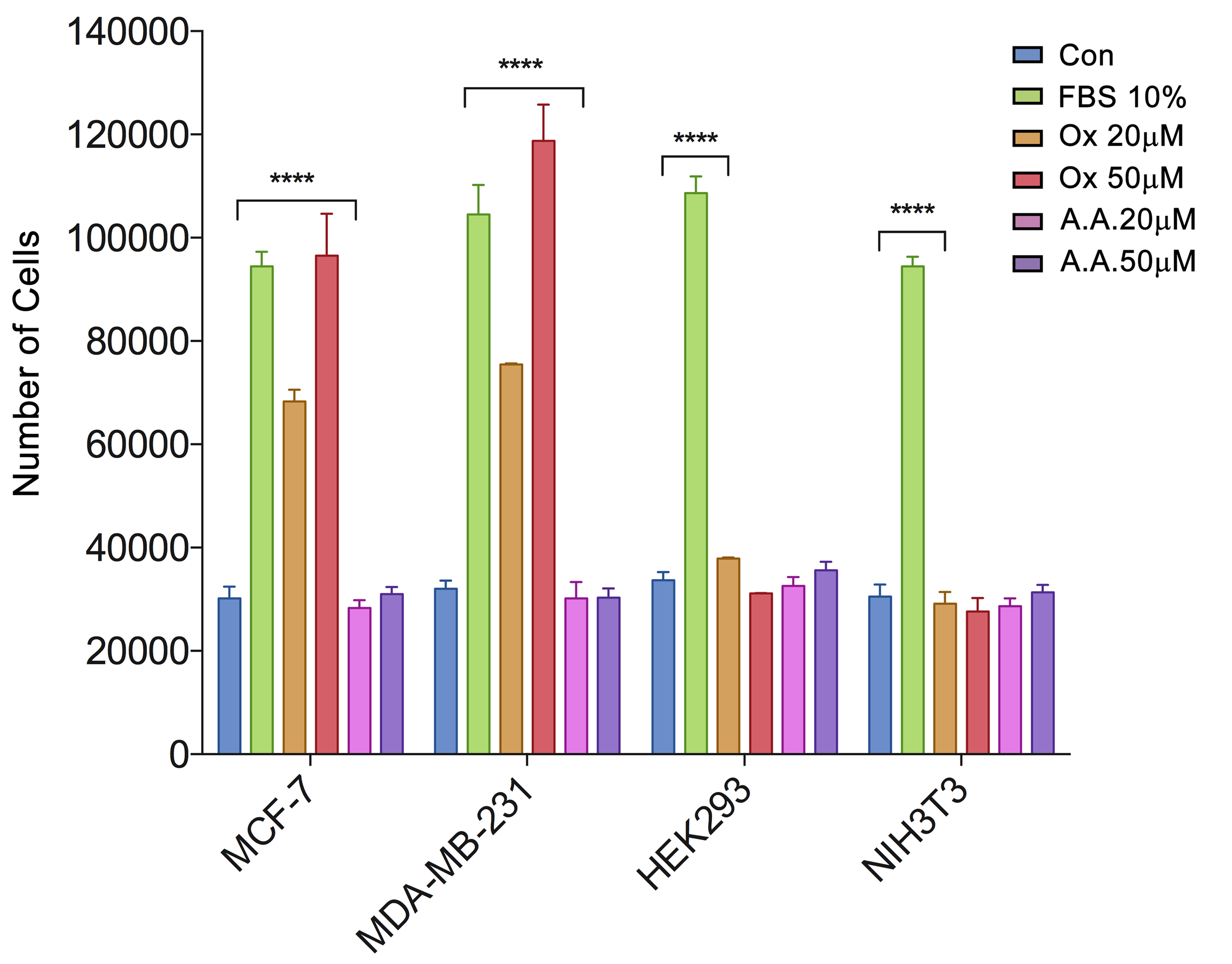

Supplement: Additional file 1: Figure S1. — Oxalate induces proliferation of breast cancer cells but not of HEK-293 and NIH-3 T3 cells. Proliferation was measured in MCF-7, MDA-MB231, HEK-293 and NIH-3 T3 cells after 3 days of treatment by counting cells in a Neubauer Chamber. Cells were cultured in DMEM medium plus an additional specific reagent or not, according to each condition. Con: Control, none additional reagent. FBS: fetal bovine serum. Ox: oxalic acid. A.A.: acetic acid. Bars represent the standard error of the mean of three independent experiments performed in triplicate. Statistical significance determined by Two-way ANOVA with Holm-Sidak’s multiple comparison test (α = 0.05) was performed in experiments graphed in Additional file 1: Figure S1. **** P < 0.0001. (TIFF 2135 kb) [file 12885_2015_1747_MOESM1_ESM.tiff]

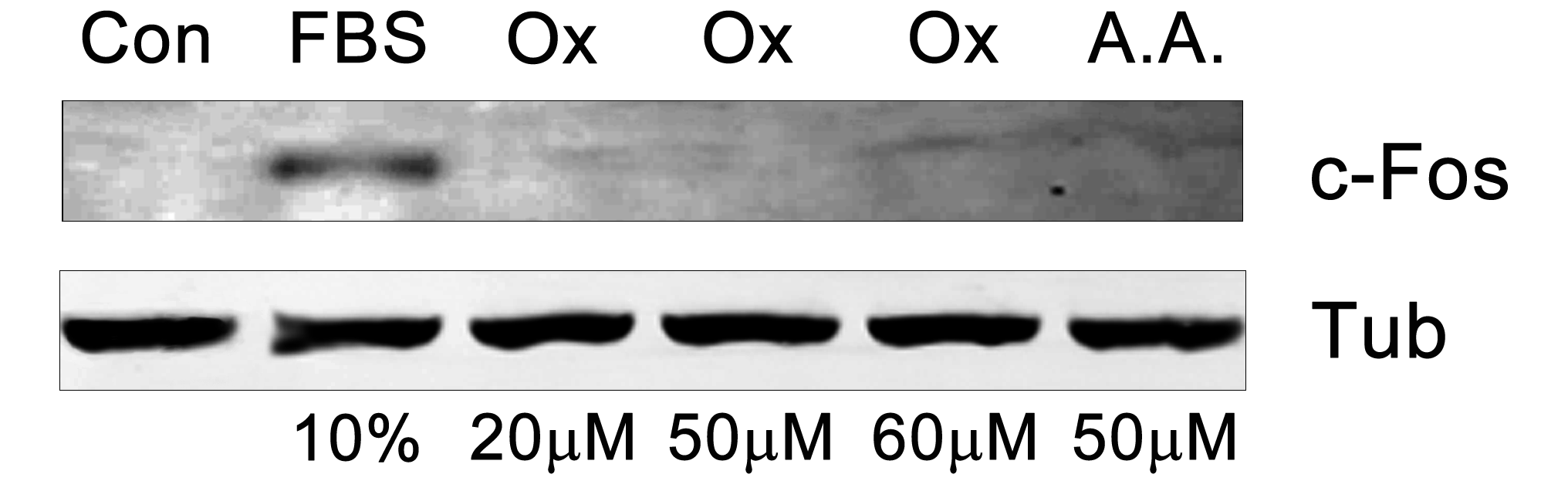

Supplement: Additional file 2: Figure S2. — Oxalate does not induce c-Fos expression in NIH-3 T3 cells. Cells were plated in six wells and growing up until 80 % of confluence. Then cells were starved to achieve quiescence (see Material & Methods). After that, cells were stimulated during 1.5 h with a specific reagent according to each condition. NIH-3 T3 cells were lysed and the supernatant fractions were separated in 12 % SDS-PAGE gel and immunoblotted using anti-c-Fos antibody. α-Tubulin was used as loading control. Con: Control, none additional reagent. FBS: fetal bovine serum. Ox: oxalic acid. A.A.: acetic acid. (TIFF 130 kb) [file 12885_2015_1747_MOESM2_ESM.tiff]

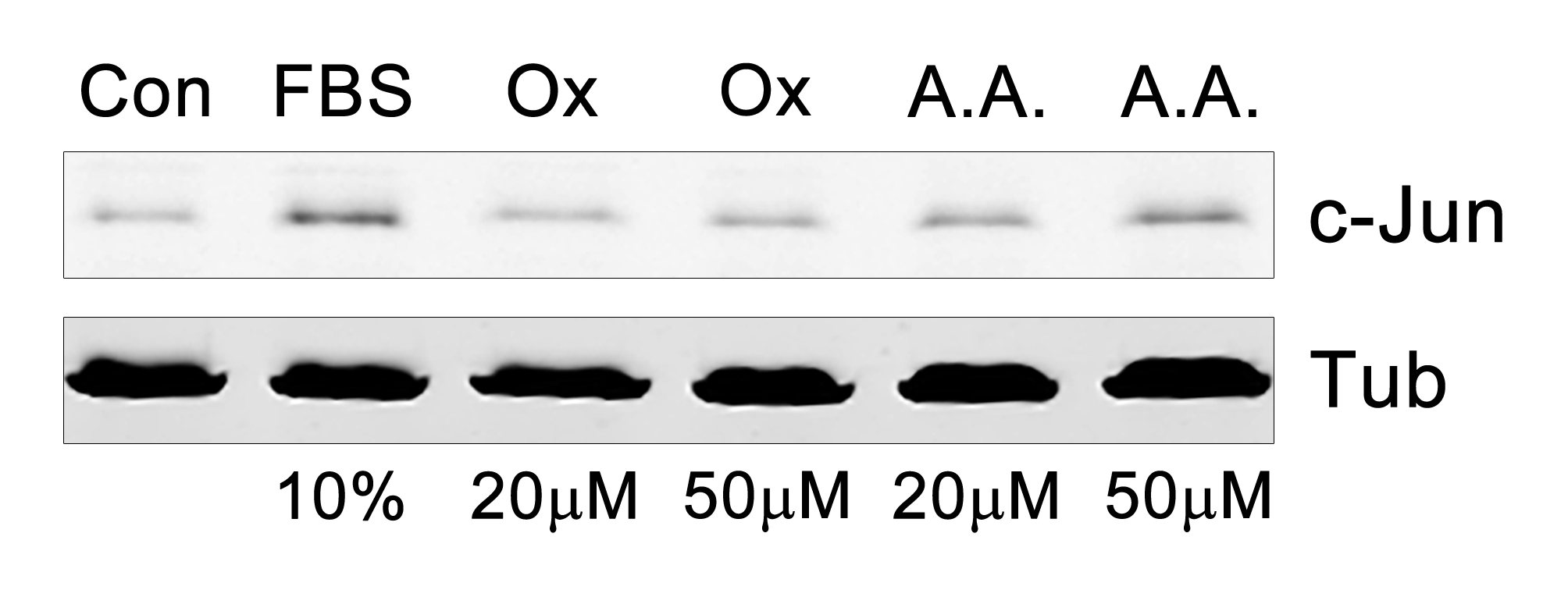

Supplement: Additional file 3: Figure S3. — Oxalate does not induce c-Jun expression in MCF-7 cells. Cells were plated in six wells and growing up until 80 % of confluence. Then cells were starved to achieve quiescence (see M & M). After that, cells were stimulated during 1.5 h with a specific reagent according to each condition. MCF-7 cells were lysed and the supernatant fractions were separated in 12 % SDS-PAGE gel and immunoblotted using anti-c-Jun antibody. α-Tubulin was used as loading control. Con: Control, none additional reagent. FBS: fetal bovine serum. Ox: oxalic acid. A.A.: acetic acid. (TIFF 132 kb) [file 12885_2015_1747_MOESM3_ESM.tiff]
